# Supplementary material for: Neurobiological Features of Posttraumatic Stress Disorder (PTSD) and Their Role in Understanding Adaptive Behavior and Stress Resilience
Source: Int J Environ Res Public Health. 2022 Aug 18;19(16):10258. doi: 10.3390/ijerph191610258 (PMC9407950; doi:10.3390/ijerph191610258)
Supplement: Supplementary file 1 [file ijerph-19-10258-s001.zip › ijerph-1839028-supplementary.pdf]

Table S1

Authors and Year	Design	n	Population	Regions of interest
Bromis K, et al. 2019 [32]	Systematic Review	6585	PTSD, NTHC, TEHC, MDD	GP, NAcc, THA, CN, TL, FPC, OFC, PHG, HPC, AM, CC, PoCG, IFG, aCC, PUT
Burkhouse K et al., 2020 [52]	Randomized clinical trial	81*	MDD, GAD	AM, NAcc, vmPFC
Douglas K et al., 2019 [49]	Case-controlled study	22	PTSD, TEHC	HPC, PHG, STG, pCC, TTG, ANG, MOG, SMG, LG, PFC, CUN, iPC
Etkin A and Wager T. 2007 [8]	Systematic review	1270	PTSD, SAD, SpPh	THA, vmPFC, aCC, mCC, pCC, AM, iPC, PCUN, PHG, IC, GP, FFG, STG, IFG, SN, PUT, dmPFC, OFC
Fitzgerald J, DiGangi J, Phan K. 2019 [15]	Narrative review	-	PTSD	aCC, AM, IC, HPC, dmPFC, dlPFC, mPFC, vmPFC
Gilbertson et al., 2002 [55]	Case-controlled study	70	PTSD	HPC
Hayes J, Hayes S, Mikedis A. 2021 [26]	Systematic review	684	PTSD, TEHC, NTHC	aCC, STG, SMA, AM, dmPFC, THA, IFG, MOG, mPFC, PCUN, CB, FFG,
Hinojosa et al., 2019 [30]	Narrative review	-	PTSD	aCC, mCC, pCC
Kasai et al., 2008 [33]	Case-controlled study	41	PTSD, NTHC	HPC, aCC, IC
Ke et al., 2017 [3]	Case-controlled study	90	PTSD, NTHC, TEHC	mCC, aCC, PCUN, mPFC, PHG, LG, OFC, AM, IC, HPC
Koch et al., 2017 [27]	Case-controlled study	77	PTSD, TEHC	Uncinate fasciculus
Morey R et al., 2021 [14]	Case-controlled study	200	PTSD, TEHC	AM, HPC

O'Doherty et al., 2015 [7]	Systematic review	846	PTSD, TEHC, NTHC***	HPC, aCC, AM
Ousdal et al., 2020 [53]	Case-controlled study	107	PTSD, NTHC	AM
Sekiguchi et al., 2012 [29]	Case-controlled study	42	TEHC	aCC, OFC
Suarez-Jimenez et al., 2019 [13]	Systematic review	304	PTSD, TEHC	AM, aCC, IC, mPFC, THA, vmPFC
Young et al., 2019 [9]	Case-controlled study	142	PTSD, TEHC	aCC
Zhang et al., 2016 [11]	Case-controlled study	57	PTSD, TEHC, NTHC	mPFC, SFG, CB, CAL, TP, CN, PCUN

Selected studies, indicting number of patients, participants' profiles and regions interest (ROI); HPC: hippocampus; AM: amygdala; aCC: anterior cingulate cortex; HPT: hypothalamus; PHG: parahippocampal gyrus; PFC: prefrontal cortex; vmPFC: ventromedial prefrontal cortex; dlPFC: dorsolateral prefrontal cortex; mPFC: medial prefrontal cortex; dmPFC: dorsomedial prefrontal cortex; vlPFC: ventrolateral prefrontal cortex; GP: globus pallidus; NAcc: accumbens nucleus; THA: thalamus; CN: caudate nucleus; M1: primary motor area/precentral gyrus; S1: primary somatosensory area/postcentral gyrus; IFGorb: inferior frontal gyrus pars orbitalis; IFGoper: IFG pars opercularis; IFGtri: IFG pars triangularis; PUT: putamen; STG: superior temporal gyrus; CAL: calcarine sulcus/fissure; TTG: transverse temporal gyrus; ANG: angular gyrus; MOG: middle occipital gyrus; SOG: superior occipital gyrus; SMG: supramarginal gyrus; LG: linguinal gyrus; CUN: cuneus; PCUN: precuneus; iPC: inferior parietal cortex; IC: insular cortex; CB: cerebellum; SFG: superior frontal gyrus; STR: striatum. MDD: major depressive disorder; SAD: social anxiety disorder; GAD: generalized anxiety disorder; PTSD: posttraumatic stress disorder; NSAD: non-specific anxiety disorder; NTHC: non-traumatized healthy controls; TEHC: trauma exposed healthy controls; PAD: panic disorders; SSD: schizophrenia spectrum disorder; SUD: substance use disorder.

** Two experiments were conducted in this study. On the second experiment with 55 patients, some of the participants were under 18 years old and therefore results were excluded the present review.*

*** Two other groups were analyzed by the author: 21 experiments involving under-age participants and 4 experiments involving participants over 50 years old. Only the experiments involving adults were considered for the present review.*

**** In 32 studies patients also presented MDD as a comorbidity (n=613); in 2 studies patients also presented BPD as a comorbidity (n=22); in 2 study patients also presented GAD as a comorbidity (n=48).*
